# Rabies virus modifies host behaviour through a snake-toxin like region of its glycoprotein that inhibits neurotransmitter receptors in the CNS

**DOI:** 10.1038/s41598-017-12726-4

**Published:** 2017-10-09

**Authors:** Karsten Hueffer, Shailesh Khatri, Shane Rideout, Michael B. Harris, Roger L. Papke, Clare Stokes, Marvin K. Schulte

**Affiliations:** 1 Department of Veterinary Medicine, University of Alaska Fairbanks, Fairbanks, Alaska United States of America; 2 Department of Pharmaceutical Sciences, University of the Sciences, Philadelphia, Pennsylvania United States of America; 3 Department of Biology and Wildlife & Institute of arctic Biology, University of Alaska Fairbanks, Fairbanks, Alaska United States of America; 4 Department of Biology, California State University Long Beach, Long Beach, California United States of America; 5 Department of Pharmacology & Therapeutics University of Florida, Gainesville, Florida United States of America

## Abstract

Rabies virus induces drastic behaviour modifications in infected hosts. The mechanisms used to achieve these changes in the host are not known. The main finding of this study is that a region in the rabies virus glycoprotein, with homologies to snake toxins, has the ability to alter behaviour in animals through inhibition of nicotinic acetylcholine receptors present in the central nervous system. This finding provides a novel aspect to virus receptor interaction and host manipulation by pathogens in general. The neurotoxin-like region of the rabies virus glycoprotein inhibited acetylcholine responses of α4β2 nicotinic receptors *in vitro*, as did full length ectodomain of the rabies virus glycoprotein. The same peptides significantly altered a nicotinic receptor induced behaviour in *C. elegans* and increased locomotor activity levels when injected into the central nervous system of mice. These results provide a mechanistic explanation for the behavioural changes in hosts infected by rabies virus.

## Introduction

Many infectious agents can affect the central nervous system of the host leading to altered host behaviour^1^. A variety of direct and indirect mechanisms have been proposed to explain parasite driven alteration of host behaviour^1^. Detailed molecular mechanisms and adaptive significance of behavioural changes induced by pathogens in the hosts are typically not well known, and experimental evidence to support these hypotheses is often lacking^2^. Rabies virus infection often causes drastic behavioural changes and is of significant public health importance as it still lacks satisfactory treatment options^3^. The manipulation hypothesis states that infectious agents cause behavioural changes in the host to favour their transmission to a another susceptible host^4^. However, the adaptive ability to manipulate the host is often not easily distinguished from general sickness behaviours^4^. Multiple hypotheses have been suggested to explain how parasitic infections specifically alter host behaviour, ranging from specific structural damage of key areas of the central nervous system to immune pathology^2^. Rabies is a disease characterized by drastic behavioural changes and neurological disorders with a case fatality rate approaching 100% in humans. Despite this, pathological changes in the brain are generally very mild, such that the causes of severe behavioural change have not been well understood. Our poor understanding of the pathogenesis likely contributes to unsatisfactory treatment options for patients infected with this relatively neglected disease despite the fact that it still kills in excess of 50,000 people annually^3^.

Nicotinic acetylcholine receptors (nAChR) were the first rabies virus receptors described^5^. A short region in the ectodomain of the rabies virus glycoprotein, with homology to some snake toxins, binds to the orthostatic binding site on muscle nAChRs^6^ and selectively binds neuronal cells^7^. Previous studies have described the functional interaction of the same neurotoxin-like viral peptide with muscle nAChRs as an assay for binding in the context of cell infection^8^. The homology between this domain of the rabies glycoproteins with that of snake toxins suggests that both protein families bind similar domains of nAChRs. The snake venom neurotoxins are strong inhibitors of nAChR and have been used to explore the function of these receptors^9,10^. The activity of these toxins further suggests the potential for interaction of rabies glycoprotein with CNS nAChRs. Disruption of nAChR function in the CNS could play an important role in rabies pathogenesis including modification of host behaviour. Since past studies have focused primarily on muscle nAChRs, little is known about the interaction of rabies virus with nAChRs in the central nervous system. nAChR are pentameric molecules that are made up of different subunits and subunit expression varies between different tissues. While α1β1δε or α1β1δγ receptors are prevalent at the neuromuscular junction, α4β2 and α7 are the most common nAChR subtypes in the CNS. Different subtypes of nAChR have distinct pharmacological properties. In this study we explored the interaction of the RGP neurotoxin-like domain and α4β2 nAChR as well the effects on animal behaviour of this interaction. As the major finding of this study we provide for the first time a molecular mechanism of behavioural modification by pathogens, using rabies as a well-known example with public health relevance.

## Results

### Binding of neurotoxin-like peptide to homologues of the extracellular domain of nAChR

**Figure 1:**
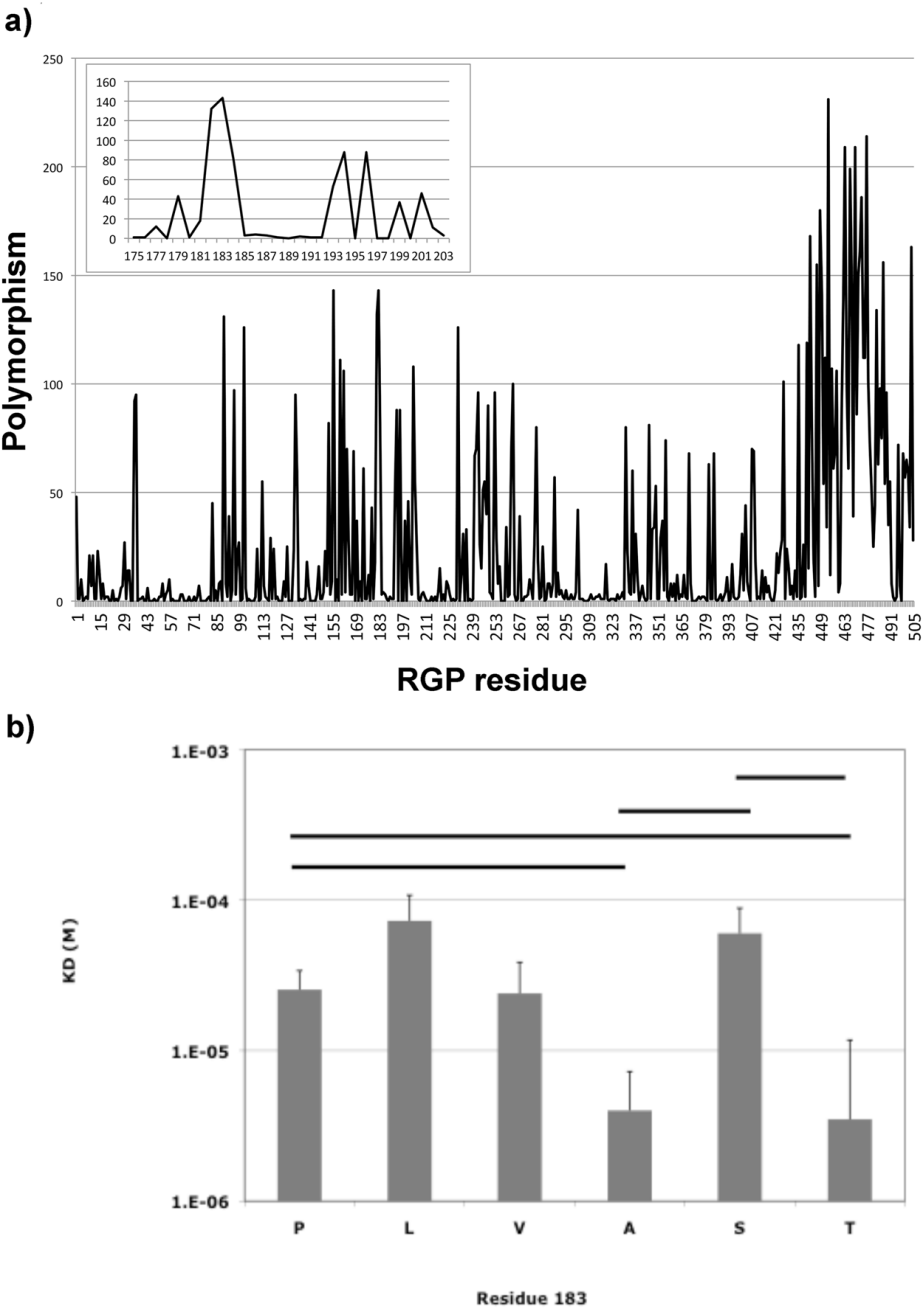
Neurotoxin-like peptides of the rabies virus glycoprotein contain polymorphic and highly conserved residues (**a**) and differ in binding to homologue of extracellular part of nAChR (**b**). (**a**) Polymorphism of RGP residues: 2649 mature RGP sequences downloaded from Genbank were submitted to the VIPR website and the polymorphism was determined and plotted over the length of the mature RGP. Residue 183 is the most polymorphic of all residues within the ectodomain (residues 1–440 in the mature RGP) of the protein. The inset represents the neurotoxin-like peptide domain used in this study. (**b**) Comparison of K_D_ values for rabies derived peptides binding to *L*-AChBP determined by Surface Plasmon Resonance. *L*-AChBP was immobilized on Biacore CM5 sensor chips and perfused with test peptides at a flow rate of 45 ul/min at 25 degrees centigrade. Average K_D_ values for at least three independent experiments and standard deviations are shown. Bars indicated significant differences between K_D_ values of two peptides as determined by Student’s T-test.

We used surface plasmon resonance (SPR) techniques to identify the interactions between neurotoxin-like peptides of the RGP and an acetylcholine binding protein derived from *Lymnaea* stagnalis (*L-AChBP*). The *L-AChBP* is a soluble pentameric homolog of the extracellular binding domain of nAChRs and has been used extensively in the study and modelling of these receptors^11–14^. Six peptides were used in the SPR assays differing only in the amino acid present at a position corresponding to residue 183 of the mature rabies glycoprotein. This residue has previously been identified as being positively selected in natural rabies isolates^15,16^ although this positive selection was not found in other studies^17^. In addition, we determined the polymorphism of residues in over 2600 glycoprotein sequences using the Virus Pathogen Resource (VIPR). Residue 183 is the most polymorphic site in the ectodomain of the rabies glycoprotein while other residues of the neurotoxin-like region are highly conserved among natural rabies viruses (Fig. 1a). A phenotype for mutations at this residue is not known. Rabies virus-derived peptides bound to *L-AChBP* with dissociation constants (K_D_) in the micromolar (μM) range, similar to those observed in previous radioligand binding studies using muscle nAChRs^6^. We detected significant differences in binding to the *L-AChBP* with K_D_ values ranging from 3.5 to 73 μM for peptides that differed only at the amino acid corresponding to residue 183 (Fig. 1b). This suggests that this residue is involved in interactions with nicotinic receptors.

### Functional effect of neurotoxin-like peptide on α4β2 nAChR

**Figure 2:**
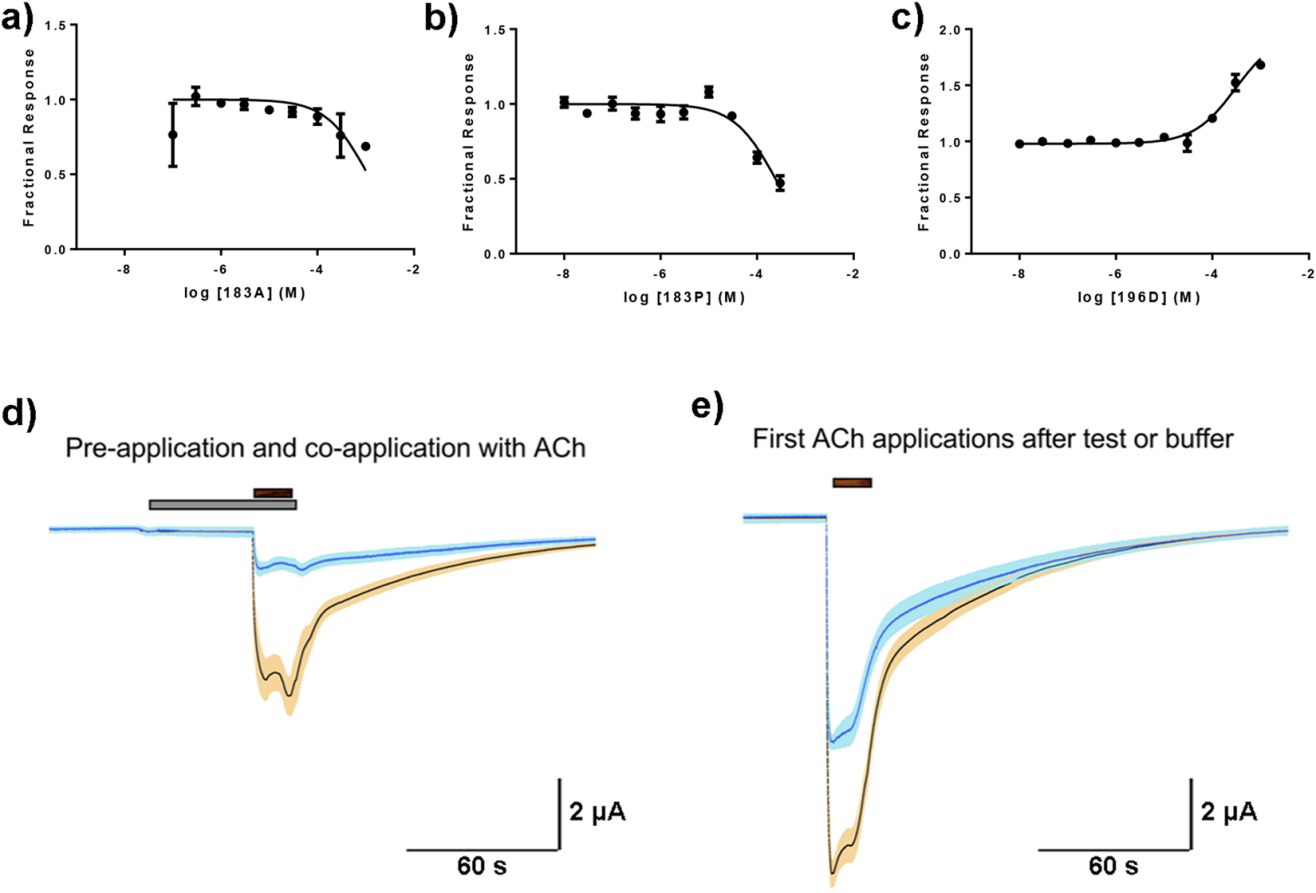
Neurotoxin-like domain of rabies virus inhibits nicotinic receptors. Functional effects of rabies glycoprotein neurotoxin-like peptides on α4β2 nAChRs (**a**–**c**). Peptides were identical in sequence with the exception of the amino acid present at position 183 (**a**,**b**) or 196 (**c**). Plots shown in (**a**,**b**) show inhibition of acetylcholine-induced responses following pre-exposure to peptides containing either a alanine (**a**) or proline (**b**) at position 183. Plot (**c**) shows the effect of a peptide containing an aspartate at position 196 (RV-R196D). The effects of full length ectodomain is shown in panels (**d,e**). Panel (d) shows the response of oocytes expressing α4β2 nAChRs to co-applications of 30 µM acetylcholine 30 seconds after pre-application of buffer (orange) or RGP ectodomain (blue) at 840 nM concentration. Panel (e) shows the recovery of responses to 30 µM acetylcholine 4 minutes after exposure to RGP ectodomain.

Next, we performed functional assays on the predominant nAChR subtype in the CNS (α4β2) to determine whether rabies neurotoxin-like region altered the activity of this critical receptor. Assays used two-electrode voltage clamp on nAChRs expressed in *Xenopus* oocytes. To limit use of vertebrate animals and to focus on the most relevant peptides, we selected the peptides with the two most common amino acids at residue 183 (alanine, and proline), which also had significantly different binding characteristics to ACHBP. A peptide with an alanine at position 183 (RV-183A) inhibited acetylcholine evoked responses on α4β2 nAChR with an extrapolated IC_50_ = 465–2620 μM at the 95% CI (Fig. 2a). A peptide with proline at this position (RV-183P) showed slightly higher potency on α4β2 receptors with an extrapolated IC_50_ of 185–314 µM (95% CI) (Fig. 2b). The differences between the relative binding to ACHBP and inhibition of α4β2 nAChR between the two peptides are likely due to differences in structure between *L-AChBP* and the α4β2 subtype of nAChR. In contrast to these two peptides, another variant (RV-R196D, with aspartic acid at position 196) produced no inhibition but instead potentiated responses to acetylcholine (extrapolated EC_50_ = 149–605 μM at the 95% CI). However, that peptide did not activate receptors on its own and so is not likely acting as an agonist. Amino acid 196 is an arginine or lysine in naturally occurring RGPs, which corresponds to the quaternary amino group in the acetylcholine molecule and therefore RV-R196D is expected to have reduced activity on the nAChR^6^. While IC_50_ concentrations in the mid micromolar range are higher than those typical of inhibitory ligands at nicotinic receptors *in vitro*, the limited volume of the synaptic cleft^18^ results in similar concentrations *in vivo* based on the estimated 1200 glycoprotein molecules present on a single virus particle^19^. Indeed, the severity of disease in mice is related to the level of glycoprotein expressed^20^, indicating that only high glycoprotein concentrations can cause the changes seen. In addition, the relatively small molecular flexibility of the intact glycoprotein as compared to that of the small peptide will likely increase the affinity of the intact protein to the nAChR. To test this, we also assessed the effects of full length RGP-ectodomain on α4β2 receptors. The ectodomain inhibited the receptors at a concentration of 840 nM (Fig. 2d,e). After the application of ACh in the presence of buffer, α4β2 control ACh responses recovered to a level not significantly different (p = 0.168 in a paired t-test, n = 5) from the ACh control responses prior to the buffer application. ACh peak currents were 11.5 ± 1.6 µA and 10.9 ± 1.1 µA prior to and after the buffer application, respectively. However, ACh control responses after the application of the rabies protein (Fig. 2e) were significantly reduced (p = 0.029 in a paired t-test, n = 6) compared to ACh controls obtained prior to the protein application. ACh peak currents were 9.4 ± 3.1 µA and 5.7 ± 1.6 µA prior to and after the protein application, respectively. These results indicate that the neurotoxin-like region, in the context of the full-length protein, has activity at even lower concentrations. Glutamate receptors were not inhibited by the ectodomain at 100 nM while this concentration still significantly inhibited α4β2 receptors compared to buffer treated receptors (data not shown).

### Behaviour modifications by neurotoxin-like peptide

**Figure 3:**
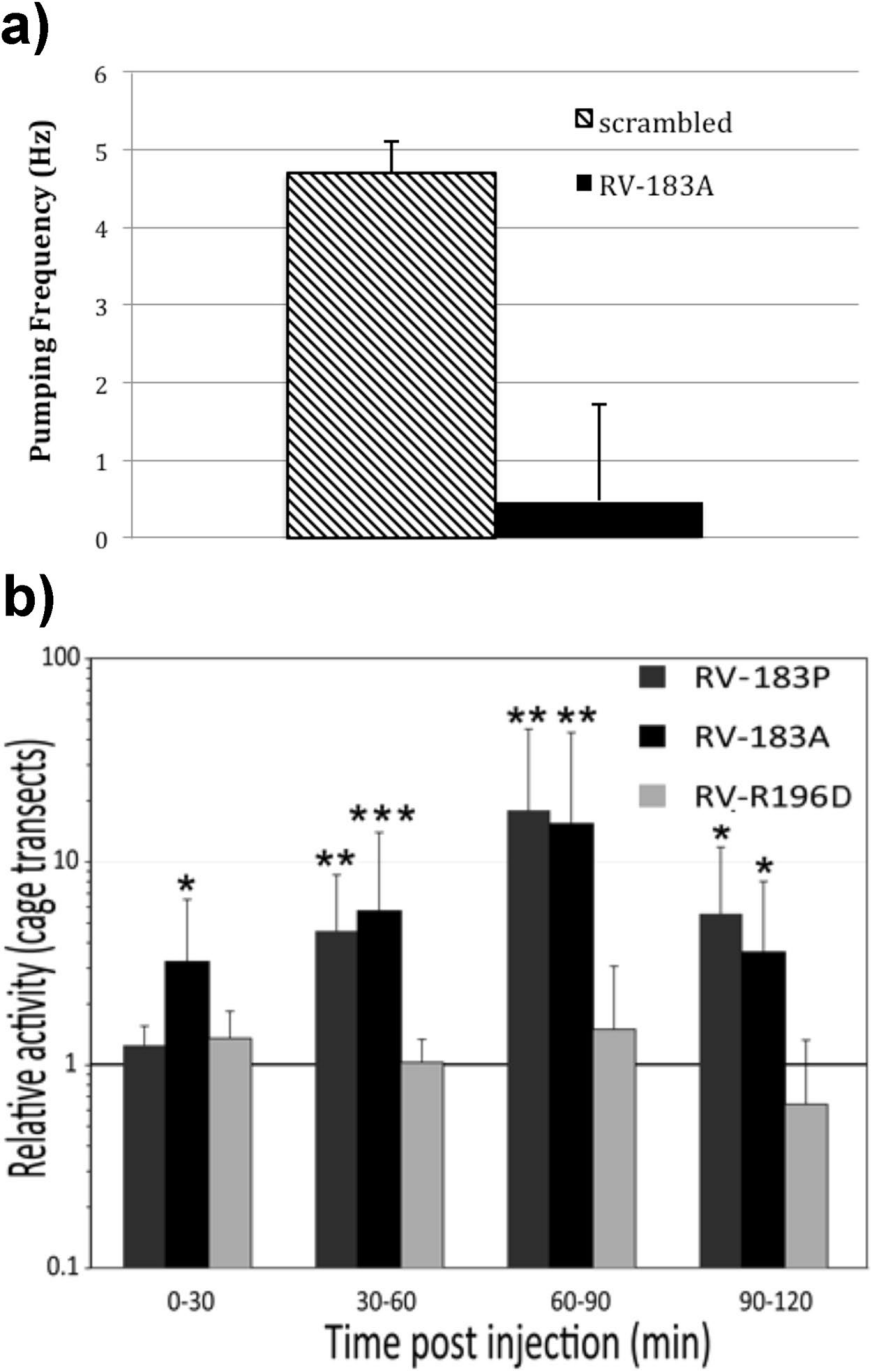
Rabies neurotoxin-like peptide effects on behaviour. (**a**) Rabies neurotoxin-like peptide inhibits the frequency of nAChR-meditated pharyngeal pumping in *C. elegans*. *C. elegans* were injected with peptide and pharyngeal pumping was measured, p < 0.001 by student-T test. (**b**) Rabies neurotoxin-like peptides alter the behaviour of mice. Mice injected with the peptide were observed and the number of cage transects were determined relative to control injected mice, the horizontal line at 1 represents control injected animals. *Indicate significance at p < 0.1 (*), p < 0.05 (**) or p < 0.01 (***) using two-way ANOVA test. A representative video of injected mice can be seen in supplemental movie.

Altered neurotransmitter receptor function could change behaviour in infected hosts. We used the neurotoxin-like peptides instead of full-length ectodomain as the ectodomain could lead to other interactions within the animal that could counteract or alter the nAChR mediated effect. To test the ability of rabies neurotoxin-like peptide to alter nAChR mediated behaviour in an established model organism *in vivo* we injected *Caenorhabditis elegans* with neurotoxin-like RPG peptides or peptides with a scrambled sequence and measured pharyngeal pumping. Pharyngeal pumping behaviour in *C. elegans* is triggered by ACh binding to the nAChR EAT-2^21^. Pharyngeal pumping was absent in six out of seven worms injected with rabies neurotoxin-like peptides (RV-183A). The remaining worm pumped intermittently leading to significantly reduced average pumping frequency (Fig. 3a).

To test if the RGP neurotoxin-like peptides have effects on behaviour in mammals we injected neurotoxin-like peptide into the cerebrospinal fluid of adult mice to reach an approximate final concentration of 250 micromolar. Mice, with rabies virus-derived peptides (RV-183A or RV-183P) injected into the lateral ventricle, showed increased locomotor activity in an novel-environment behaviour test, when compared to mice receiving control peptide (identical amino acid composition but with a scrambled sequence) or saline (Fig. 3 and supplemental material). Hyperactivity is one clinical sign of rabies^22^. The peptide RV-R196D that did not inhibit α4β2 nAChR signalling also did not alter mouse behaviour in this assay, showing that behavioural influences of these peptides are related to specific interactions with the nAChR.

## Discussion

This study offers a new look at host pathogen interactions in the context of behavioural modification as outlined in the manipulation hypothesis^4^. Interactions of the neurotoxin-like region of rabies virus glycoprotein lead to behaviour modifications through inhibition of CNS nAChR. A small region of the glycoprotein inhibits a nAChR-initiated behaviour in *C. elegans* and induces a rabies-associated behaviour in mice. We provide a novel mechanistic explanation for behavioural manipulation of host animals by pathogens, through inhibition of neurotransmitter receptors. Similar manipulations of host behaviour are found in many pathogen-host interactions but have so far lacked a satisfactory mechanistic explanation.

Our findings align with studies that showed reduced locomotor activity in mice after nicotine administration mediated by α4β2 nAChR receptors^23^. Furthermore, impaired serotonin neurotransmitter function has been implicated in the pathogenesis of rabies including the behavioural changes induced by the virus^24^. Activation of presynaptic α4β2 receptors increases excitability of serotonin neurons through glutamate release in the dorsal raphe nuclei^25^. The inhibition of α4β2 receptors by the RGP demonstrated in this study could therefore influence the behaviour of infected animals through alteration of serotonin function as well.

No detailed structural model exists for the rabies virus glycoprotein at this time. However, the region corresponding to neurotoxin-like peptide of rabies virus is exposed on the surface of the glycoprotein in a model of the VSV G protein^26^. Furthermore, this region of the virus is part of the major epitope II of the rabies virus glycoprotein^27^, indicating that it is accessible to antibody binding in the mature glycoprotein and may interact with the nAChR or other neuroreceptors in the CNS of infected animals. Our experiments show that the RGP neurotoxin-like peptide inhibited α4β2 nAChR subtype in the mid-micromolar range. These values are higher than those typical of inhibitory ligands at nicotinic receptors. However, the limited volume within the synaptic cleft of 0.76 × 10 − 3 µm^3^ ^18^ would result in *in vivo* concentrations of approximately 2.5 mM from the estimated 1200 glycoprotein molecules present on a single virus particle^19^. In addition, *in vitro* studies have shown that the glycoprotein is released from the surface of infected cells^28^, possibly further increasing accessibility of the neurotoxin like domain to interact with nAChRs in the synapse. Therefore the concentrations used in this study may be biologically relevant, at the presumed site of activity, the synaptic cleft.

Traditionally, virus-receptor interaction and host influence is thought to be due to effects on viral cell entry and immune recognition. The present findings demonstrate that this may be expanded to include specific neurotransmitter receptor based manipulation of the CNS of infected hosts. This insight presents a new, testable hypothesis about the complexity of pathogen-host interactions and opens the possibility of using these interactions to probe the function of the CNS using viral and possibly other parasitic mechanism. In addition, for diseases such as rabies that currently lack a satisfactory therapy once symptoms occur, our results suggest new approaches by inhibiting the neurological activity of virus-derived peptides in the CNS.

## Methods

### Peptides and proteins

The peptides were synthesized at Elim Biopharmaceuticals. Sequences of all peptides used in this study are provided in Table 1. The ectodomain sequence was based on the RGP sequence from a rabies isolate obtained from a red fox in Ontario (Genbank Accession AAA65971). This is a naturally occurring rabies strain that causes behavioural modification in its natural host. The protein was expressed in insect cells using a baculovirus system by Protein Science (Meriden, Connecticut).

**Table 1:** Peptides used in this study, Amino acids at residues corresponding to 183 and 196 in the mature rabies glycoprotein are bolded and underlined.

RV-183P	YTIWMPEN**P**RLGTSCDIFTNS**R**GKRASKG
RV-183A	YTIWMPEN**A**RLGTSCDIFTNS**R**GKRASKG
RV-183L	YTIWMPEN**L**RLGTSCDIFTNS**R**GKRASKG
RV-183V	YTIWMPEN**V**RLGTSCDIFTNS**R**GKRASKG
RV-183S	YTIWMPEN**S**RLGTSCDIFTNS**R**GKRASKG
RV-183T	YTIWMPEN**T**RLGTSCDIFTNS**R**GKRASKG
RV-R196D	YTIWMPEN**P**RLGTSCDIFTNS**D**GKRASKG
Scrambled	GSEPKWYRTKDRASPGSNFTITLMGRCNI

### Determining the level of polymorphisms in RGP

RGP sequences (partial and full length) were downloaded from Genbank on 12/11/2013. Sequences of vaccine strains and other artificial constructs and sequences not covering the region of the neurotoxin-like region were discarded. The remaining 2649 sequences were submitted to the Virus Pathogen Resource database and normalized entropy of the observed allele distribution was determined for residue using the following formula: S = −100 * Sum (Pi * log_2_Pi); where Pi is the frequency of the ith allele.

### Surface Plasmon Resonance

All SPR experiments were performed using a Biacore 2000 instrument and CM5 research grade sensor chips. A 100 mM phosphate buffer pH 7.4 containing 150 mM NaCl and 0.005% surfactant P-20, was used as a running buffer for all experiments. Synthetic *Lymnaea* stagnalis AChBP was produced from stably transfected HEK-293 cells (CRL-1573, American Type Culture Collection) using a synthetic cDNA inserted into a p3xFLAG-CMV-9 vector and containing a C-terminal 6x Histidine region and an N-terminal p3-Flag epitope tag. Using standard Biacore protocols, the *L-AChBP* was covalently linked to the CM5 sensor chip. Typical immobilization levels were 1500–5000 resonance units (RU). Test peptides at 3.125 µM to 100 µM in running buffer were introduced at a flow rate was 45 ul/min and allowed to disassociate for 10 minutes. At least one control injection containing only running buffer (no peptide) was placed between each injection of a sample peptide as a control. Typically multiple control samples were run before and after peptide injections to ensure a stable response. SPR data were analysed using BIAevaluation software, version 3.0. Kinetic rate constants are globally determined by fitting the biosensor data using numerical integration and nonlinear least squares analysis. In cases were on/off rates are too rapid to determine, Kd values were determined by plotting the plateau of the SPR response versus ligand concentration.

### Electrophysiology

For the experiments evaluating the neurotoxin-like domain:

*Xenopus* laevis oocytes were prepared as previously described and microinjected with 50 nl 0.2–0.3 ng/µl synthetic α4β2 receptor cRNAs (50%/50% ratio of α and β subunits)^29^. Recordings were made using conventional two-electrode voltage clamp in vertical flow profusion chambers as described^30^. Oocytes were exposed to increasing concentration of peptides ranging from 0.01 to 1000 µM. Concentrations in excess of 1 mM were not used due to potential non-specific effects. Peptides showing antagonist properties were evaluated by pre-exposing the oocyte to the antagonist for 30 s prior to introduction of acetylcholine. No peptide tested was capable of activating a receptor in the absence of acetylcholine. Peak currents were measured and normalized to the maximum response obtained on exposure to acetylcholine at a concentration equal to its EC90. Dose response Curves were fit using Prism version 5.0 (GraphPad Software Inc., San Diego, CA.). Since complete inhibition curves were not possible, the top and bottom values of each curve were fixed at 1 and 0 respectively and the Hill slope was fixed at 1.0 to facilitate curve fitting. Values shown in Fig. 2 are extrapolated estimates of the IC_50_ and EC_50_ values for each of the three peptides tested and are given as the 95% confidence intervals.

For the experiments evaluating the full length ectodomain:

The human α4 and β2 nAChR clones were obtained from Dr. Jon Lindstrom (University of Pennsylvania, Philadelphia PA). Oocytes were surgically removed from mature *Xenopus laevis* frogs (Nasco, Ft. Atkinson WI) and injected with the nAChR subunit cRNAs at a 1:1 ratio, as described previously^31^.

Two-electrode voltage clamp experiments were conducted using OpusXpress 6000 A (Molecular Devices, Union City, CA)^31^. Both the voltage and current electrodes were filled with 3 M KCl. Oocytes were voltage-clamped at −60 mV. The oocytes were bath-perfused with Ringer’s solution (115 mM NaCl, 2.5 mM KCl, 1.8 mM CaCl_2_, 10 mM HEPES, and 1 μM atropine, pH 7.2) at 4 ml/min. For the purpose of normalization, two control responses to 30 µM ACh were obtain prior to the application of the experimental buffer with or without the rabies protein. Control responses were defined as the average of these two initial applications of ACh made before test applications. The solutions were applied from a 96-well plate via disposable tips. ACh applications were 6 s in duration followed by a 241 s washout period. Buffer with or without the rabies protein was pre-applied for 30 s before the co-application of buffer with ACh.

### Microinjection of peptide into *C. elegans*

Approximately 50 pl of 5 mM of either a 29 amino acid glycoprotein peptide fraction or a scrambled peptide (control) was pressure injected across the cuticle proximal to the pharynx of N2 wild type *C. elegans* worms using standard methods^32^. Following injection, worms were incubated with 10 mM serotonin to enhance pharyngeal pumping and pump frequency was determined from the electropharyngeogram (EPG) using a ScreenChip system (NemaMetrix, Eugine, OR.)^33^.

### Mouse behavioural assays

Mice were chronically instrumented with a re-entry cannula into the lateral ventricle (26 gauge, C315G; Plastics One, Roanoke VA)^34,35^. After recovery for at least 7 days, mice were anesthetized using isoflurane while 2 microliter of peptide (at 5 mM) was injected over 2 seconds into the lateral ventricle to reach a concentration of approximately 250 micromolar in the cerebrospinal fluid. Mice were then observed by video camera for 4 hours within a novel enclosure (20 cm × 41 cm). Activity levels of mice receiving rabies virus derived peptides were assessed by counting enclosure transects and compared to those of mice that received control peptide (scrambled) or saline solution over 30 minute intervals. Results were analysed to determine differences between mice injected with rabies-derived peptides and control injected mice using two way ANOVA for each time period. No difference was detected between PBS and scrambled peptide injected animals.

### Ethics Statement

All experiments were done in accordance with the guidelines of the Guide for the Care and Use of Laboratory Animals of the National Institutes of Health and were approved by the University of Alaska Fairbanks Institutional Animal Care and Use Committee of the University of Alaska Fairbanks (protocol # 347492-1).

## Electronic supplementary material

Supplementary Movie

Movie caption
