# Supplementary material for: Rabies virus modifies host behaviour through a snake-toxin like region of its glycoprotein that inhibits neurotransmitter receptors in the CNS
Source: Sci Rep. 2017 Oct 9;7:12818. doi: 10.1038/s41598-017-12726-4 (PMC5634495; doi:10.1038/s41598-017-12726-4)
Supplement: Supplementary file 2 — Movie caption [file 41598_2017_12726_MOESM2_ESM.doc]

Rabies virus modifies host behaviour through a snake-toxin like region of its glycoprotein that inhibits neurotransmitter receptors in the CNS.

Karsten Hueffer^1*^, Shailesh Khatri^2^, Shane Rideout^3^, Michael B. Harris^3^ ^¶^ , Roger L. Papke^4^, Clare Stokes^4^, Marvin K. Schulte^2^

**Supplementary movie:**

Increased locomotive behaviour of mice injected with rabies neurotoxin-like peptide.

The video shows a three minute interval of a behaviroural assay about 1 hour post injection on peptide into the lateral ventricle. The mouse on the left received rabies derived peptide (RV-183P) and the mouse on the left received scrambled peptide. The video is sped up 3 fold.
